# On modelling the relationship between vegetation greenness and water balance and land use change

**DOI:** 10.1038/s41598-018-27139-0

**Published:** 2018-06-13

**Authors:** Sandra L. Berry, Brendan Mackey

**Affiliations:** 1 Environmental Futures Research Institute, Griffith University, Gold Coast City, Australia; 2 Griffith Climate Change Response Program, Griffith University, Gold Coast City, Australia

## Abstract

Here we sought a biologically meaningful, climate variable that captures water-energy availability and is suitable for high resolution (250 m × 250 m) modelling of the fraction of photosynthetically active radiation intercepted by the sunlit canopy (*F*_*V*_) derived from a 10-year (July 2000 – June 2010) time series of Moderate Resolution Imaging Spectroradiometer (MODIS) Normalized difference vegetation index (NDVI) satellite imagery for Australia. The long-term mean annual evaporation deficit, and mean annual water availability indices all yielded strong linear relationships with mean *F*_*V*_ ($\overline{{{\boldsymbol{F}}}_{{\boldsymbol{V}}}}$, %). We hypothesised whether some of the scatter about the relationships was related to land-use changes that have disrupted the vegetation-climate-soil equilibrium. Using continental-scale spatial data layers of protected area status and vegetation condition classes we repeated our analyses with restricted datasets. $\overline{{{\boldsymbol{F}}}_{{\boldsymbol{V}}}}$ of intact native vegetation within protected areas was greater than all modified vegetation classes. There was a consistent decline in the slopes of the regression relationships with increasing intensity of woody vegetation clearing and livestock grazing. Where native vegetation has been transformed by land use there was a 25% reduction in predicted $\overline{{{\boldsymbol{F}}}_{{\boldsymbol{V}}}}$.

## Introduction

Two widely accepted certainties in life are that the climate of the Earth is changing, and that the concentration of carbon dioxide in the atmosphere is increasing. These two factors directly impact on vegetation growth and cover: climate change impacts on the supply of water for transpiration that necessarily occurs as plants take up CO_2_ from the atmosphere for photosynthesis, and; the CO_2_ concentration directly affects the water use efficiency of photosynthesis^1^. Other factors that also impact on photosynthesis, plant growth and canopy cover include soil properties and nutrient availability, sunlight and disturbances. Over most of the terrestrial surface, provided climatic conditions are suitable for plant growth, the indigenous vegetation has had thousands of years to adapt to post-Last Glacial Maximum soil properties and light availability. Disturbance by humans through land clearing, land-use change, changed fire regimes and the introduction of non-indigenous grazing animals, however, is on-going. How this disturbance impacts on annual to multi-year scale greenness of vegetation cover is uncertain, as it is difficult to quantify at the continental scale.

In this paper we have two interrelated goals: to identify a robust, biologically meaningful, climate variable that can be used for high resolution modelling of vegetation greenness under potential future climate scenarios, and; to use available spatial data layers to investigate continental-scale impacts of land-clearing and land-use change on vegetation. Our measure of vegetation greenness is the percentage of photosynthetically active radiation intercepted by the sunlit canopy averaged over the annual, or decadal period, $\bar{{F}_{v}}$, where the overbar indicates the mean value. *F*_*v*_ as defined here is equivalent to the term FAPAR which has been shown to be correlated with the density of green chloroplasts (hence vegetation greenness), photosynthesis and thus gross primary productivity (the rate of new biomass production)^2,3^. *F*_*v*_ is not to be confused with similar terms used in the study of vegetation florescence^4^. Our study area is the Australian continent. Australia is large (7,659,861 km^2^, comparable to the area of conterminous USA), mega-diverse with some 147,579 formally described species and an estimated total of over 550,000 species^5^, together with a rich diversity of bioregions, landscapes and vegetation ecosystems spanning tropical through temperate climates (arid to moist), and a 200-year history of contemporary land use and land use change^6^. We derive a continental 10-year time-series of $\overline{{F}_{v}}$ at 250 m spatial resolution from Moderate Resolution Imaging Spectroradiometer (MODIS) Normalized difference vegetation index (NDVI) satellite imagery satellite imagery^7^.

## Identifying Candidate Climate Parameters

As water-energy availability is a major driver of plant growth and vegetation cover^1,8^ we investigate the utility of selected climate wetness parameters for predicting *F*_*v*_. Several schemes have previously been used to map climatic climax vegetation at broad geographical scales, in terms of natural vegetation classes, based on correlations with a range of climatic parameters. The parameters selected have historically been constrained by the availability of climate data and have been assessed according to their capacity to correctly classify the physiognomy of the North American vegetation. Inputs into these models have included actual evaporation (*E*; the sum of evaporation from transpiration, interception and soil), potential evaporation (*E*_*P*_), evaporation deficit (*D; D* = *E*_*P*_ − *E*), minimum temperature (T_min_), precipitation (*P*) and precipitation surplus (*S; S* = *P* − *E*). The combinations of *E* and *D*^9^ and *E* and *E*_*P*_^10^ have been found to be the most successful predictors of vegetation class. These parameters are biologically meaningful and readily interpreted, coupling together the water and heat balances of the land surface.

*E*_*P*_ is a measure of evaporative demand, more formally defined as the rate of evaporation that will occur from a well-watered, actively growing, green sward completely covering the ground surface^10^. *E*_*P*_ may be calculated from measured pan evaporation (*E*_*pan*_), multiplied by a constant term known as the pan coefficient (*k*, for most countries *k* = 0.70, for Australia k = 0.75)^11^. Where measurements are not available, *E*_*P*_ is generally calculated from a formula. Several formulations for the estimation of evaporation parameters have been developed^8,11–14^ but their utility for spatial analyses is contingent on the availability of suitable spatial data layers of input variables including the net radiation flux (*R*_*N*_). The formulation for *E*_*P*_ requiring the fewest input variables is that of Budyko^8^;

1 $${E}_{P}\cong \frac{{R}_{N}}{\lambda }$$

where λ is the latent heat of vaporisation of water (~2.5 × 10^6^ J kg^−1^ H_2_O).

As spatial estimates of *R*_*N*_ for Australia were not available, Berry and Roderick^15,16^ calculated estimates of an annual measure of water availability ($\overline{{W}_{Q}}$ mm yr^−1^), substituting available spatial estimates of solar radiation incident at the surface over a year ($\overline{{Q}_{s}}$, J m^−2^ yr^−1^);

2 $$\overline{{W}_{Q}}=\bar{P}-\overline{{Q}_{s}}/\lambda $$

where *W*_*Q*_ indicates that the evaporation term of the water availability index is calculated from solar radiation ($\overline{{Q}_{s}}/\lambda$), and $\bar{P}$ is the average annual precipitation (mm yr^−1^). (The overbar indicates that the parameter is the long-term mean value). $\overline{{Q}_{s}}/\lambda$ represents the depth of water that would be evaporated from a plane surface of unit area if all of the solar radiation incident at the surface was utilized for evaporation. In reality, that is not the case as a fraction of *Q*_*s*_ is reflected and some of the remaining *Q*_*s*_ is converted to sensible heat.

When potential evaporation data is available an alternative estimate of the annual water availability is $\overline{{W}_{E}}$ (mm yr^−1^);

3 $${\bar{W}}_{E}=\bar{P}-\overline{{E}_{P}}$$

$\bar{W}$ ($\overline{{W}_{E}}$ or $\overline{{W}_{Q}}$) can be expected to have predictive capacity similar to the evaporation deficit, $\bar{D}$. By assuming that the change in soil water storage over the annual and longer periods is zero, the water balance of a grid cell (as the spatial unit of analysis) can be simplified to:

4 $$\bar{P}=\bar{E}+\bar{r}$$

where $\bar{r}$ is the net runoff. When $\bar{E}$ is limited by the supply of $\bar{P}$, (i.e., water-limited environments) $\bar{r}\cong \,0$ and $\bar{P}\cong \bar{E}$. As $\overline{{Q}_{s}}/\lambda$ is a surrogate for $\overline{{E}_{P}}$:

$$\bar{W}\approx \bar{E}-\overline{{E}_{P}}$$

and;

$$\bar{W}\approx -\bar{D}$$

In environments where $\bar{E}$ is limited by the supply of energy (i.e., energy-limited environments), $\bar{W}$ has a positive value equal to the surplus precipitation, whilst $\bar{D}$ equals zero.

Berry and Roderick^16^ found that $\overline{{W}_{Q}}$ was strongly correlated with $\overline{{F}_{V}}$ estimated from a 10-year time-series of monthly NDVI images (NOAA AVHRR, ~8 km grid cell size, resampled to 0.05° × 0.05° to match the climate datasets). In that study $\overline{{F}_{V}}$ increased more or less linearly with $\overline{{W}_{Q}}$ until a threshold at approximately −1000 mm yr^−1^, representing the point of balance between precipitation and evaporation. The relationship for grid cells where evaporation was limited by the supply of water was described using the linear regression equation:

5 $$\overline{{F}_{V}}=0.99+2.78\times {10}^{-4}\overline{{W}_{Q}}\,({{\rm{R}}}^{2}=0.81)$$

Beyond the threshold (i.e. $\overline{{W}_{Q}}$> −1000 mm yr^−1^) there was no further increase in $\overline{{F}_{V}}$. Berry and Roderick^16^ demonstrated a general relationship between $\overline{{F}_{V}}$, $\overline{{W}_{Q}}$ and vegetation classes represented in the digital map of present Australian vegetation cover, mapped at a cartographic scale of 1:5 000 000^17^.

Although Berry and Roderick quantitatively demonstrated the intimate coupling between vegetation cover and productivity and environmental water and energy supply, there was considerable scatter, where scatter is defined as the difference between the observed and predicted $\overline{{F}_{V}}$. Potential causes of the scatter were not investigated as the coverage of individual grid cells at the spatial scale of the analysis was approximately 27 km^2^ and, consequently, there were numerous possible causes of variability within a cell arising from landscape heterogeneity and precipitation estimates. (No spatial data of vegetation condition for Australia were available at the time of Berry and Roderick’s analysis.) For $\overline{{W}_{Q}}$ < −1000 mm yr^−1^ it was implicitly assumed that there was no net runoff, hence water availability may have been overestimated in some cells.

Studies by Donohue and colleagues^18–20^ have investigated the relationships between vegetation cover derived from NOAA AVHRR NDVI (8 km grid cells) and water balance parameters for Australian water catchments with the aim of improving predictions of runoff and reducing scatter about the Budyko curve. As noted, the Budyko curve describes the relationship between $P/{E}_{P}$ and $E/{E}_{P}$. They found, however, that the inclusion of vegetation parameters in the hydrological models did not significantly improve the models predictive capacity.

Given the above, we chose to compare the utility of the evaporation deficit, $\bar{D}$, and the water availability measures, $\,\overline{{W}_{E}}$ and $\overline{{W}_{Q}}$ for prediction of $\overline{{F}_{V}}$, over the annual period. We hypothesised that some of the scatter (Equation 5) in the study of Berry and Roderick may be due to (1) the coarse (0.05 degree) spatial resolution of the dataset used by Berry and Roderick and (2) anthropogenic impacts on natural vegetation cover such as clearing, thinning and livestock grazing.

## Anthropogenic Impacts On Natural Vegetation Cover

There is evidence for the earliest occupation of the Australian continent by humans between 70,000 and 40,000 years ago^21,22^. Over the following millennia humans undoubtedly impacted on the vegetation directly, especially through use of fire, and indirectly, through alteration to ecosystem process such as vegetation-herbivore interactions associated with substantial and contemporaneous extinction of megafauna^23^. At the commencement of the period of occupation by European peoples about 220 years ago, which coincides with the commencement of the ‘industrial period’, the vegetation is generally considered to have been in a ‘natural’ condition^24^. Subsequently, much of Australia’s vegetation has been altered due to land-use change though some remains in a natural condition where climate, topography and/or soil properties render the land unsuitable for other uses. Major changes to vegetation structure and cover have been mapped at the continental scale^24,25^. Additionally, spatial data layers having continental coverage of protected area status^26^ and vegetation condition^27^ provide information about land-use and land-cover.

Theory of optimality of projected foliage cover of natural vegetation under existing annual average water balance was proposed by Eagleson^28^. His model demonstrated that a stable-equilibrium exists between water-limited natural vegetation systems and their climate and soil environments when the canopy density and species act to minimise water stress. Diversity of species within natural vegetation cover allows for many possible ways in which the photosynthetic tissues comprising the canopy can be arranged in space and time. These arrangements, or *ecological strategies*^29^ allow individual plants or species to persist at a site. Annual and ephemeral plants produce leaves and carry out photosynthesis, hence contribute to *F*_*V*_, when there is sufficient moisture and energy in the upper soil profile. They have short-lived leaves which die when the soil dries out. The deciduous strategy is similar but woody stems persist from year to year and woody roots allow these plants to access water at greater soil depth. Leaves of plants utilizing the annual/ephemeral and the deciduous strategies are capable of high rates of photosynthesis and thus high rates of transpiration. Lower rates of photosynthesis and lower rates of water-use are typical of evergreen woody, perennial herbaceous and cacti/succulent strategies. The increased leaf-longevity arises from the presence of a higher proportion of structural tissue relative to photosynthetic tissue in the leaves. In water-limited environments evergreen woody trees have very extensive root systems and are able to access water several meters below the soil surface. In order to survive dry periods, perennial herbaceous plants and some woody evergreens that obtain their water requirements from the upper soil profile make conservative use of water.

Land use change may result in replacement of natural vegetation with other vegetation that utilises the same ecological strategy and extracts water from similar depths within the soil profile, for example, the replacement of natural forest with a plantation forest. More commonly, however, land use change involves the removal of some, or all, deep-rooted natural vegetation (trees and tall shrubs), and replacement by shallow rooted strategies (annuals and ephemerals). During periods of evaporation deficit the shallow-rooted plants will desiccate and die. We hypothesise that where annual average evaporation is limited by water availability, annual average $\overline{{F}_{V}}$ of modified vegetation will be lower than that of the natural vegetation. We investigate whether this reduction in $\overline{{F}_{V}}$ arising from land-use change depicted by land condition mapping can be detected at the continental scale.

## Methods

### Vegetation parameters

To address our hypotheses, we calculated $\overline{{F}_{V}}$ from a 10-year time-series of 16-day composite MODIS satellite NDVI imagery at a spatial resolution of 250 m (9 seconds) for Australia, and gridded climate surfaces of monthly rainfall, pan evaporation and global radiation derived using a digital elevation model at the same resolution. In order to capture the range of between year variability of $\overline{{F}_{V}}$ we calculated $\bar{<mml:mpadded xmlns:xlink="http://www.w3.org/1999/xlink" voffset="0">{F}_{VMIN}</mml:mpadded>}$ and $\bar{<mml:mpadded xmlns:xlink="http://www.w3.org/1999/xlink" voffset="0">{F}_{VMAX}</mml:mpadded>}$, being the minimum and maximum mean annual *F*_*V*_ over the 10-year time-series. Further details on the methods used to calculate $\overline{{F}_{V}}$ and derive the climate surfaces are given in Additional Methods. For the estimation of *F*_*V*_ we did not assume that the baseline soil NDVI has a constant value over all soil types. Instead, we constructed a spatial data layer of NDVI_soil_ by combining readily available surface lithology mapping and an analysis, for individual pixels within each major lithology group, of minimum NDVI values. For further details, see Additional Methods in Supplementary Information.

### Climate parameters

Gridded surfaces of monthly rainfall, pan evaporation and global radiation were generated using the ANUCLIM software package^30^ at a spatial scale commensurate with the vegetation data (see Additional Methods in Supplementary Information). The water availability indices $\overline{{W}_{Q}}$ and $\overline{{W}_{E}}$ were calculated using Eqs 2 and 3 (see above).

$\overline{{E}_{P}}$ was calculated from spatially extrapolated measurements of pan evaporation, $\bar{<mml:mpadded xmlns:xlink="http://www.w3.org/1999/xlink" voffset="0">{E}_{pan}</mml:mpadded>}$, where $\bar{<mml:mpadded xmlns:xlink="http://www.w3.org/1999/xlink" voffset="0">{E}_{p}</mml:mpadded>}=0.75\bar{<mml:mpadded xmlns:xlink="http://www.w3.org/1999/xlink" voffset="0">{E}_{pan}</mml:mpadded>}$.

The empirical formulation of Choudhury^31^ was used to produce spatial data layers of $\bar{E}$;

6 $$\bar{E}=\frac{\bar{P}}{{[1+{(\frac{\bar{P}}{\overline{{E}_{P}}})}^{\propto }]}^{1/\propto }}$$

where α is a ‘catchment properties parameter’ that alters the partitioning of *P* to *E*_*P*_ and runoff. A range of values of α were applied to calculate $\bar{D}$;

7 $$\bar{D}=\overline{{E}_{P}}-\bar{E}$$

The value of α selected for analyses was that which produced the nearest to linear relationship with $\overline{{F}_{V}}$ (see below).

### Vegetation – climate relationship

The relationship between $\overline{{F}_{V}}$ and $\bar{D}$ was investigated through linear least squares regression analyses. The dataset for the initial analyses included all vegetated pixels over the continent. As an aim of this study was to assess the predictive capacity of environmental variables, we attempted to minimize the signal of random environmental events (for example, drought, wildfire and cyclones) by calculating the maximum annual value $\bar{<mml:mpadded xmlns:xlink="http://www.w3.org/1999/xlink" voffset="0">{F}_{vmax}</mml:mpadded>}$. It has previously been shown that the coefficient of variance (COV) in *F*_*V*_ of natural vegetation in undisturbed environments is small^32^. In that case $\bar{<mml:mpadded xmlns:xlink="http://www.w3.org/1999/xlink" voffset="0">{F}_{vmax}</mml:mpadded>}\cong \bar{<mml:mpadded xmlns:xlink="http://www.w3.org/1999/xlink" voffset="0">{F}_{V}</mml:mpadded>}$.

Spatial data analyses were performed using ARCGIS 10.1^33^ and IDRISI Taiga^34^ geographic information system software. Pixels having a cover of mangroves, inland freshwater, salt lakes and lagoons, sea and estuaries, and dunes, as mapped by the National Vegetation Information System (DSEWP 2012) and the Surface Geology of Australia^35^ were excluded from the analysis.

To investigate possible causes of scatter about the regression relationships, we examined the relationships between $\overline{{F}_{V}}$, and $\bar{D}$, $\bar{<mml:mpadded xmlns:xlink="http://www.w3.org/1999/xlink" voffset="0">{F}_{vmax}</mml:mpadded>}$ and $\bar{D}$, and the COV of $\overline{{F}_{V}}$ and anthropogenic impacts on natural vegetation cover using two spatial data sets with continental coverage. The first data set – the Collaborative Australian Protected Areas Database (CAPAD) – delineated the boundaries of protected areas within the national reserve system^26^. We assumed that the conservation status of the land tenure was associated with minimal, or at least lesser, impacts by modern land use activities such as mining, logging and pastoralism. The second data set, called VAST^36^, provided continental mapping of land areas allocated into ranked classes that indicate the relative extent to which the natural vegetation condition had been degraded by land use impacts: (1) Residual (native vegetation intact, i.e., not significantly perturbed from land use/land use management practice); (2) Modified (native vegetation retains community structure, composition and regenerative capacity but has been perturbed by land use/land management practice); (3) Transformed (native vegetation community structure, composition and regenerative capacity significantly altered by land use/land management practice); (4) Replaced – adventive, (native vegetation replaced by alien species with spontaneous occurrence); (5) Replaced – managed (native vegetation replacement with cultivated species); and (6) Removed (vegetation removed). As categories 4 and 6 were represented by 0% and 0.1% of the study area they were excluded from the analyses.

## Results

### Relationship between $\overline{{{\boldsymbol{F}}}_{{\boldsymbol{V}}}}$ and water availability indices, $\bar{<mml:mpadded xmlns:xlink="http://www.w3.org/1999/xlink" voffset="0">{{\boldsymbol{W}}}_{{\boldsymbol{Q}}}</mml:mpadded>}$ and $\bar{<mml:mpadded xmlns:xlink="http://www.w3.org/1999/xlink" voffset="0">{{\boldsymbol{W}}}_{{\boldsymbol{E}}}</mml:mpadded>}$

**Figure 1:**
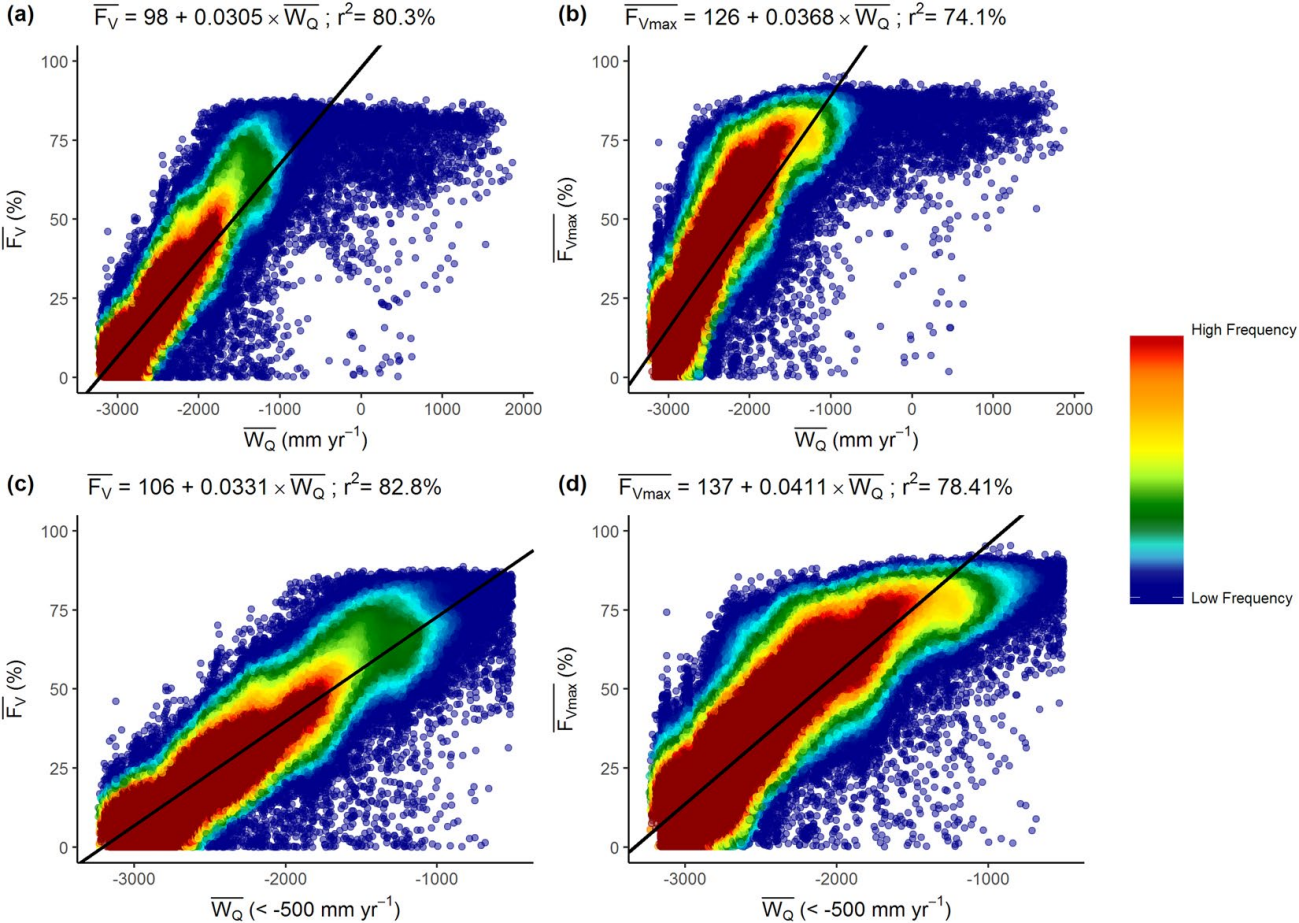
Relationship between the water availability index, $\bar{<mml:mpadded xmlns:xlink="http://www.w3.org/1999/xlink" voffset="0">{W}_{Q}</mml:mpadded>}$ (mm yr^−1^) and (**a**) $\overline{{F}_{V}}$ (**b**) $\bar{<mml:mpadded xmlns:xlink="http://www.w3.org/1999/xlink" voffset="0">{F}_{vmax}</mml:mpadded>}$ calculated using Eq. 3. (**c**) $\overline{{F}_{V}}$ and $\bar{<mml:mpadded xmlns:xlink="http://www.w3.org/1999/xlink" voffset="0">{W}_{Q}</mml:mpadded>}$ (<−500, mm yr^−1^); (**d**) $\bar{<mml:mpadded xmlns:xlink="http://www.w3.org/1999/xlink" voffset="0">{F}_{vmax}</mml:mpadded>}$ and $\bar{<mml:mpadded xmlns:xlink="http://www.w3.org/1999/xlink" voffset="0">{W}_{Q}</mml:mpadded>}$ (<−500, mm yr^−1^).

The MODIS satellite derived estimates of $\overline{{F}_{V}}$ and $\bar{<mml:mpadded xmlns:xlink="http://www.w3.org/1999/xlink" voffset="0">{F}_{vmax}</mml:mpadded>}$ increased almost linearly with $\bar{<mml:mpadded xmlns:xlink="http://www.w3.org/1999/xlink" voffset="0">{W}_{Q}</mml:mpadded>}$, until a threshold of −1500 mm yr^−1^ for the upper-, and −500 mm yr^−1^ for the lower boundary of $\overline{{F}_{V}}$ and $\bar{<mml:mpadded xmlns:xlink="http://www.w3.org/1999/xlink" voffset="0">{F}_{vmax}</mml:mpadded>}$ (Fig. 1a,b). Beyond these thresholds evaporation is limited by energy availability and there is no relationship between the parameters. Pixels in energy limited environments ($\bar{<mml:mpadded xmlns:xlink="http://www.w3.org/1999/xlink" voffset="0">{W}_{Q}</mml:mpadded>}$>−500, mm yr^−1^) comprise just 0.55% of all pixels, and consequently their exclusion had little impact on the regression relationship (Fig. 1c,d).

**Figure 2:**
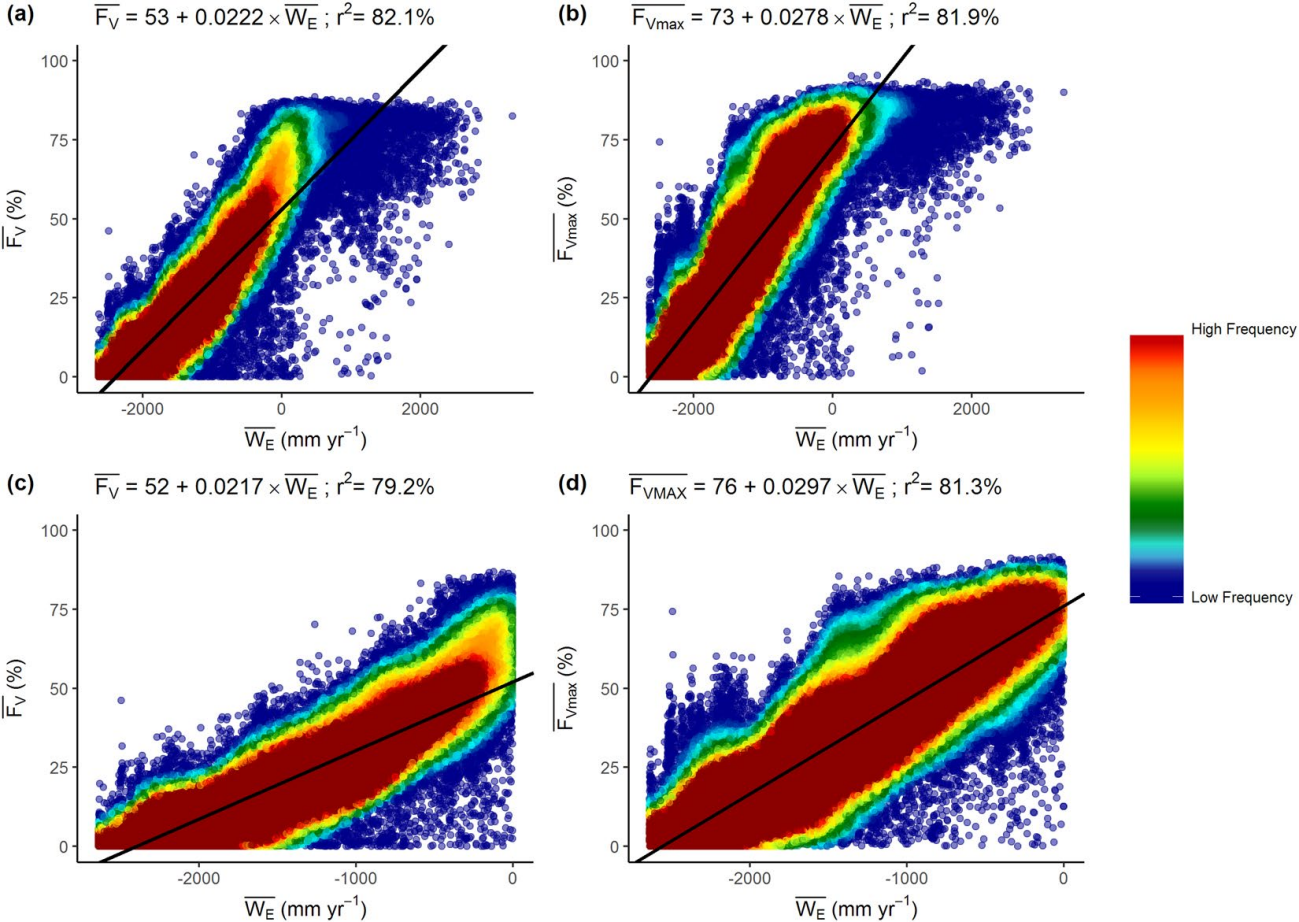
Relationship between the water availability index, $\bar{<mml:mpadded xmlns:xlink="http://www.w3.org/1999/xlink" voffset="0">{W}_{E}</mml:mpadded>}$ (mm yr^−1^) and (**a**) $\overline{{F}_{V}}$, (**b**) $\bar{<mml:mpadded xmlns:xlink="http://www.w3.org/1999/xlink" voffset="0">{F}_{vmax}</mml:mpadded>}$ calculated using Equation 3; (c) $\overline{{F}_{V}}$ and $\bar{<mml:mpadded xmlns:xlink="http://www.w3.org/1999/xlink" voffset="0">{W}_{E}</mml:mpadded>}$ (<0, mm yr^−1^); (**d**) $\bar{<mml:mpadded xmlns:xlink="http://www.w3.org/1999/xlink" voffset="0">{F}_{vmax}</mml:mpadded>}$ and $\bar{<mml:mpadded xmlns:xlink="http://www.w3.org/1999/xlink" voffset="0">{W}_{E}</mml:mpadded>}$ (<0, mm yr^−1^).

There is a slightly stronger relationship between MODIS satellite derived estimates of $\overline{{F}_{V}}$ and $\bar{<mml:mpadded xmlns:xlink="http://www.w3.org/1999/xlink" voffset="0">{F}_{vmax}</mml:mpadded>}$ and $\bar{<mml:mpadded xmlns:xlink="http://www.w3.org/1999/xlink" voffset="0">{W}_{E}</mml:mpadded>}$, (Fig. 2a,b). The point of water balance ($\overline{{E}_{P}}-\bar{P}=0$ mm yr^−1^) coincides with the threshold of the upper boundary of both $\overline{{F}_{V}}$ parameters. Least squares regression analysis accounts for 82% of the covariance of $\overline{{F}_{V}}$ and $\bar{<mml:mpadded xmlns:xlink="http://www.w3.org/1999/xlink" voffset="0">{F}_{vmax}</mml:mpadded>}$ with $\bar{<mml:mpadded xmlns:xlink="http://www.w3.org/1999/xlink" voffset="0">{W}_{E}</mml:mpadded>}$, however, visual analysis indicates that the regression equation in Fig. 2b better predicts F_V_ (i.e. $\bar{<mml:mpadded xmlns:xlink="http://www.w3.org/1999/xlink" voffset="0">{F}_{vmax}</mml:mpadded>}$) as $\bar{<mml:mpadded xmlns:xlink="http://www.w3.org/1999/xlink" voffset="0">{W}_{E}</mml:mpadded>}$ approaches 0 mm yr^−1^. For ~3.35% of pixels $\bar{<mml:mpadded xmlns:xlink="http://www.w3.org/1999/xlink" voffset="0">{W}_{E}</mml:mpadded>}$, and exclusion of these pixels from the analysis had little effect on the resulting regression equation.

**Figure 3:**
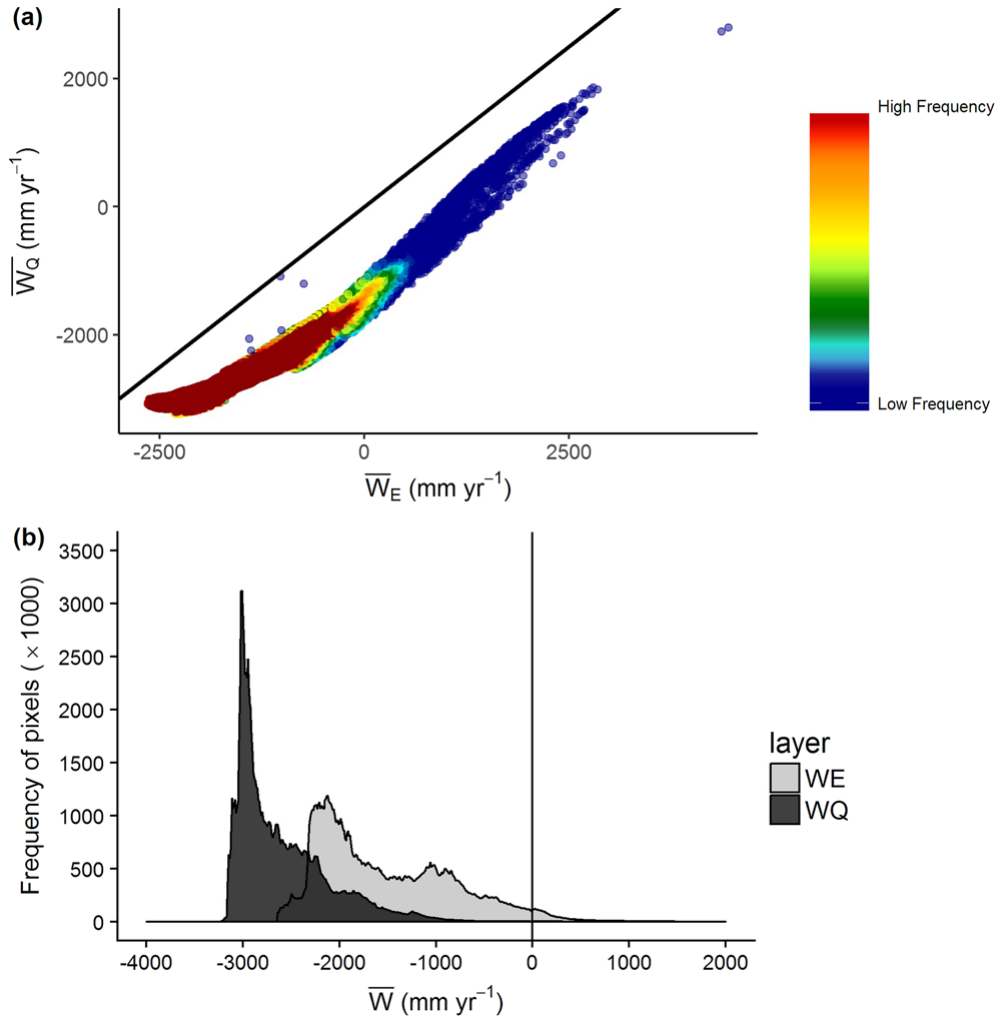
Comparison of $\bar{<mml:mpadded xmlns:xlink="http://www.w3.org/1999/xlink" voffset="0">{W}_{E}</mml:mpadded>}$ and $\bar{<mml:mpadded xmlns:xlink="http://www.w3.org/1999/xlink" voffset="0">{W}_{Q}</mml:mpadded>}$. (**a**) Relationship between $\bar{<mml:mpadded xmlns:xlink="http://www.w3.org/1999/xlink" voffset="0">{W}_{E}</mml:mpadded>}$ and $\bar{<mml:mpadded xmlns:xlink="http://www.w3.org/1999/xlink" voffset="0">{W}_{Q}</mml:mpadded>}$. Units are mm yr^−1^. The black line indicates the location of a 1:1 relationship. The equation for the regression line (not drawn) is $\overline{{W}_{Q}}=-\,1531+0.69\times \overline{{W}_{E}}$; r^2^ = 93.0. (**b**) Distribution of pixel count frequency along the gradient of increasing $\bar{<mml:mpadded xmlns:xlink="http://www.w3.org/1999/xlink" voffset="0">{W}_{E}</mml:mpadded>}$ and $\bar{<mml:mpadded xmlns:xlink="http://www.w3.org/1999/xlink" voffset="0">{W}_{Q}</mml:mpadded>}$.

Whilst $\bar{<mml:mpadded xmlns:xlink="http://www.w3.org/1999/xlink" voffset="0">{W}_{E}</mml:mpadded>}$ and $\bar{<mml:mpadded xmlns:xlink="http://www.w3.org/1999/xlink" voffset="0">{W}_{Q}</mml:mpadded>}$ are strongly correlated (Fig. 3a) there is still some ‘scatter’ about the relationship. This is expected as the radiation parameter, *Q*_*S*,_ includes energy that would be transformed to sensible heat and energy that would be reflected from the surface. $\bar{<mml:mpadded xmlns:xlink="http://www.w3.org/1999/xlink" voffset="0">{W}_{Q}</mml:mpadded>}$ values are consistently 1000 to 1700 mm yr^−1^ less than $\bar{<mml:mpadded xmlns:xlink="http://www.w3.org/1999/xlink" voffset="0">{W}_{E}</mml:mpadded>}$ (Fig. 3b).

### Relationship between $\overline{{{\boldsymbol{F}}}_{{\boldsymbol{V}}}}$ and $\bar{{\boldsymbol{D}}}$

**Figure 4:**
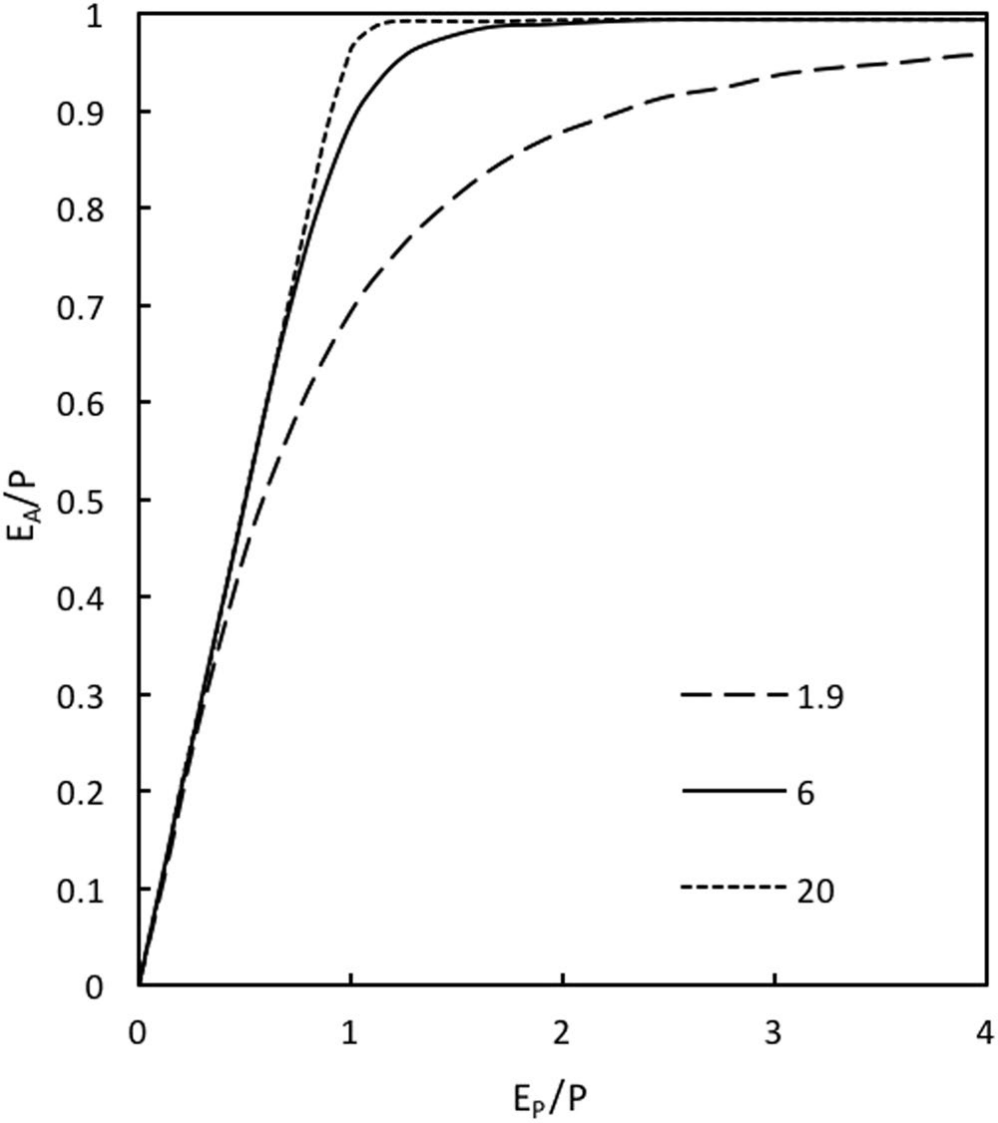
Budyko curves showing relationship between *E*_*P*_/*P* (x-axis) and *E*_*A*_/*P* (y-axis) for α = 1.9, α = 6.0, α = 20.0, (see Equation 6). Environmental conditions are water-limited when *E*_*P*_/*P* > 1 and energy-limited when *E*_*P*_/*P* < 1 In energy limited conditions the area above the line represents runoff.

**Figure 5:**
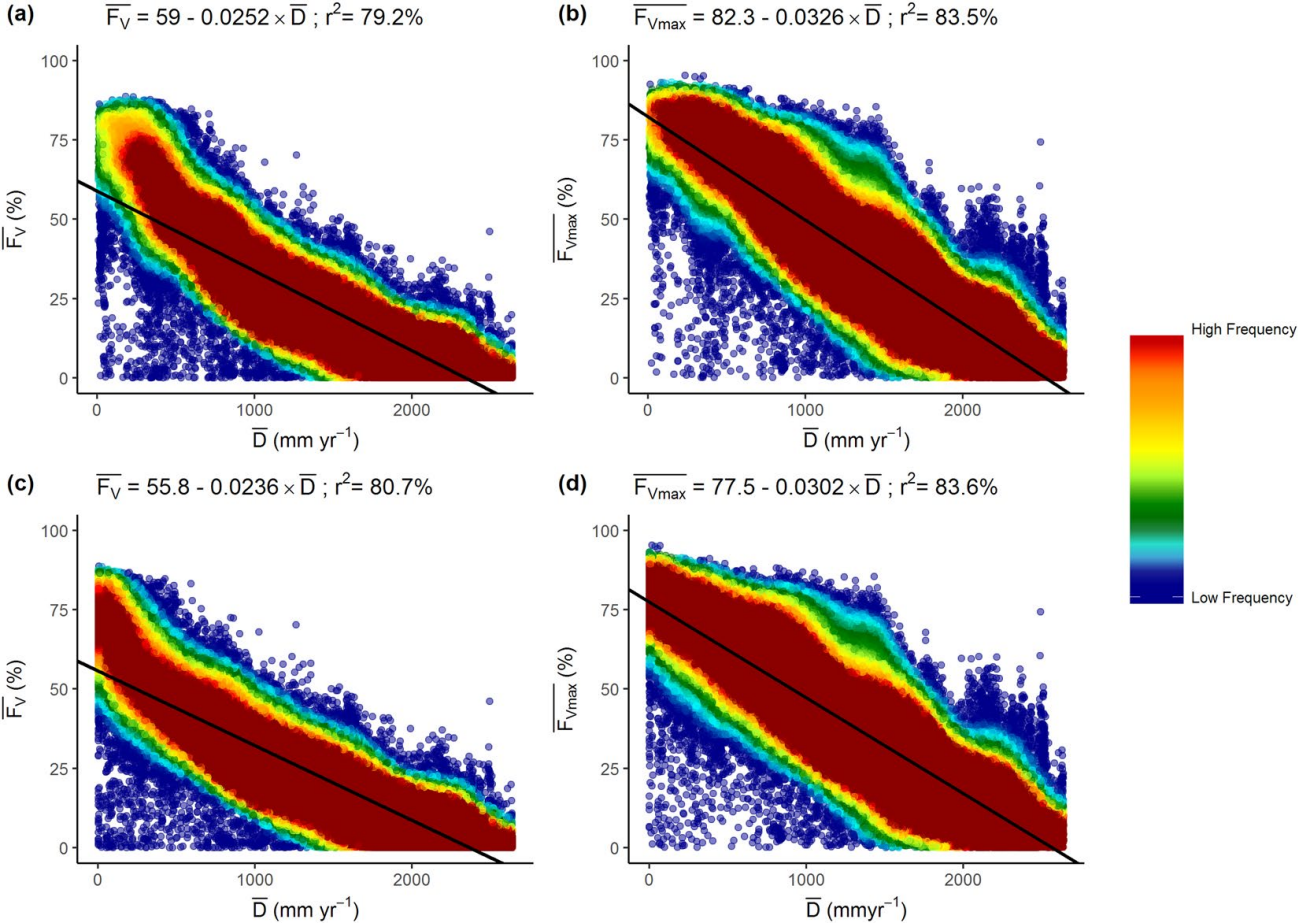
Relationship between the evaporation deficit, $\bar{D}$ (mm yr^−1^) calculated using Equation 6, and (**a**) $\overline{{F}_{V}}$, α = 1.9; (**b**) $\bar{<mml:mpadded xmlns:xlink="http://www.w3.org/1999/xlink" voffset="0">{F}_{vmax}</mml:mpadded>}$ α = 1.9; (**c**) $\overline{{F}_{V}}$, α = 6.0; (**d**) $\bar{<mml:mpadded xmlns:xlink="http://www.w3.org/1999/xlink" voffset="0">{F}_{vmax}</mml:mpadded>}$ α = 6.0.

We sought the value of α (Equation 6) that models runoff from vegetated grid cells, sufficient to result in a near-linear relationship between the evaporation deficit, $\bar{D}$ ($\bar{D}=\overline{{E}_{P}}-\overline{{E}_{A}}$, mm yr^−1^) and $\overline{{F}_{V}}$ and $\bar{<mml:mpadded xmlns:xlink="http://www.w3.org/1999/xlink" voffset="0">{F}_{vmax}</mml:mpadded>}$. Donohue and colleagues found that a value of α = 1.9 best reproduced the original Budyko curve for water catchments at 8 km spatial resolution^37^. For α = 1.9 approximately 30% of the rainfall is partitioned to runoff at the point of water-balance (ie. ${E}_{P}/P=1$). For α = 6.0 and α = 20.0 the corresponding runoff at the point of water-balance is 10% and 5% respectively (Fig. 4). For all values of α there was a strong correlation between $\bar{D}$ and $\overline{{F}_{V}}$ and $\bar{<mml:mpadded xmlns:xlink="http://www.w3.org/1999/xlink" voffset="0">{F}_{vmax}</mml:mpadded>}$. The apparently nearest to linear relationship between $\bar{D}$ and $\bar{<mml:mpadded xmlns:xlink="http://www.w3.org/1999/xlink" voffset="0">{F}_{vmax}</mml:mpadded>}$ occurred when α was assigned a value of 6.0. For smaller values of α, the relationship was curvilinear as $\bar{D}$ approaches zero. Further increases in the value of α had negligible impact on the $\bar{<mml:mpadded xmlns:xlink="http://www.w3.org/1999/xlink" voffset="0">{F}_{vmax}</mml:mpadded>}$: $\bar{D}$ relationship. The relationship between $\bar{<mml:mpadded xmlns:xlink="http://www.w3.org/1999/xlink" voffset="0">{F}_{vmax}</mml:mpadded>}$ and $\bar{D}$ with α = 1.9 and for α = 6.0 is shown in Fig. 5. A least squares regression accounts for 84% of the covariance of $\bar{<mml:mpadded xmlns:xlink="http://www.w3.org/1999/xlink" voffset="0">{F}_{vmax}</mml:mpadded>}$ with $\bar{D}$.

### Relationship between $\bar{{\boldsymbol{D}}}$ and ${\bar{{\boldsymbol{W}}}}_{{\boldsymbol{E}}}$

**Figure 6:**
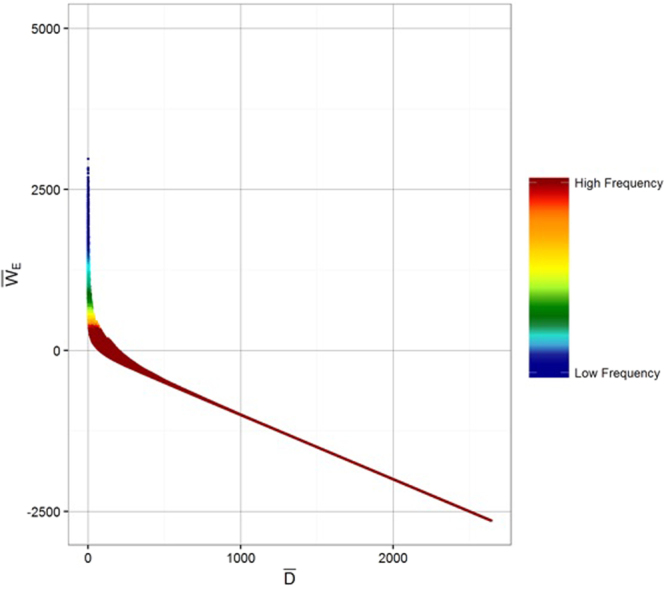
Relationship between $\bar{D}$ and $\bar{<mml:mpadded xmlns:xlink="http://www.w3.org/1999/xlink" voffset="0">{W}_{E}</mml:mpadded>}$. The scatter is due to runoff.

The same input data layers were used to calculate $\bar{D}$ and ${\bar{W}}_{E}$ and so it is not surprising that these variables are highly correlated (Fig. 6), and have similar capacity to predict $\bar{<mml:mpadded xmlns:xlink="http://www.w3.org/1999/xlink" voffset="0">{F}_{vmax}</mml:mpadded>}$. The major difference between these variables occurs with moderate to high rainfall, and is entirely attributable to partitioning to runoff (see Equation 6).

### Effects of land use change on the relationship between $\bar{<mml:mpadded xmlns:xlink="http://www.w3.org/1999/xlink" voffset="0">{{\boldsymbol{F}}}_{{\boldsymbol{v}}{\bf{m}}{\boldsymbol{a}}{\boldsymbol{x}}}</mml:mpadded>}$, $\overline{{{\boldsymbol{F}}}_{{\boldsymbol{V}}}}$, COV and $\bar{{\boldsymbol{D}}}$

**Figure 7:**
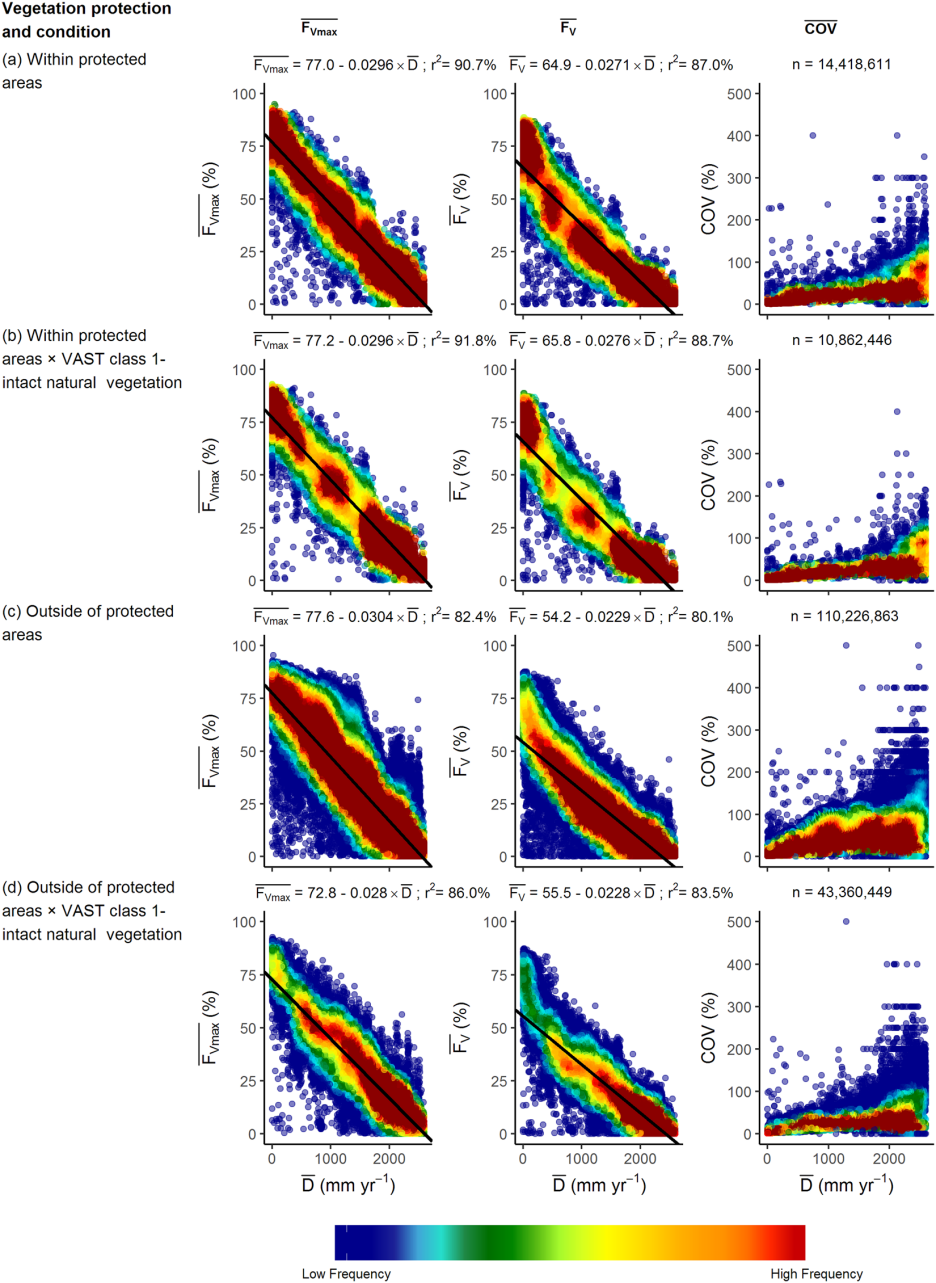
Relationship between $\bar{<mml:mpadded xmlns:xlink="http://www.w3.org/1999/xlink" voffset="0">{F}_{vmax}</mml:mpadded>}$ (%), $\overline{{F}_{V}}$ (%), COV of $\overline{{F}_{V}}$ (%) and $\bar{D}$ (mm yr^−1^) for α = 6.0 (**a**) within protected areas mapped by CAPAD 2008; (**b**) within protected areas mapped by CAPAD 2008 and having intact natural vegetation cover (VAST class 1); (**c**) outside of protected areas (Not CAPAD); (**d**) outside of protected areas and having intact natural vegetation cover (VAST class 1).

**Figure 8:**
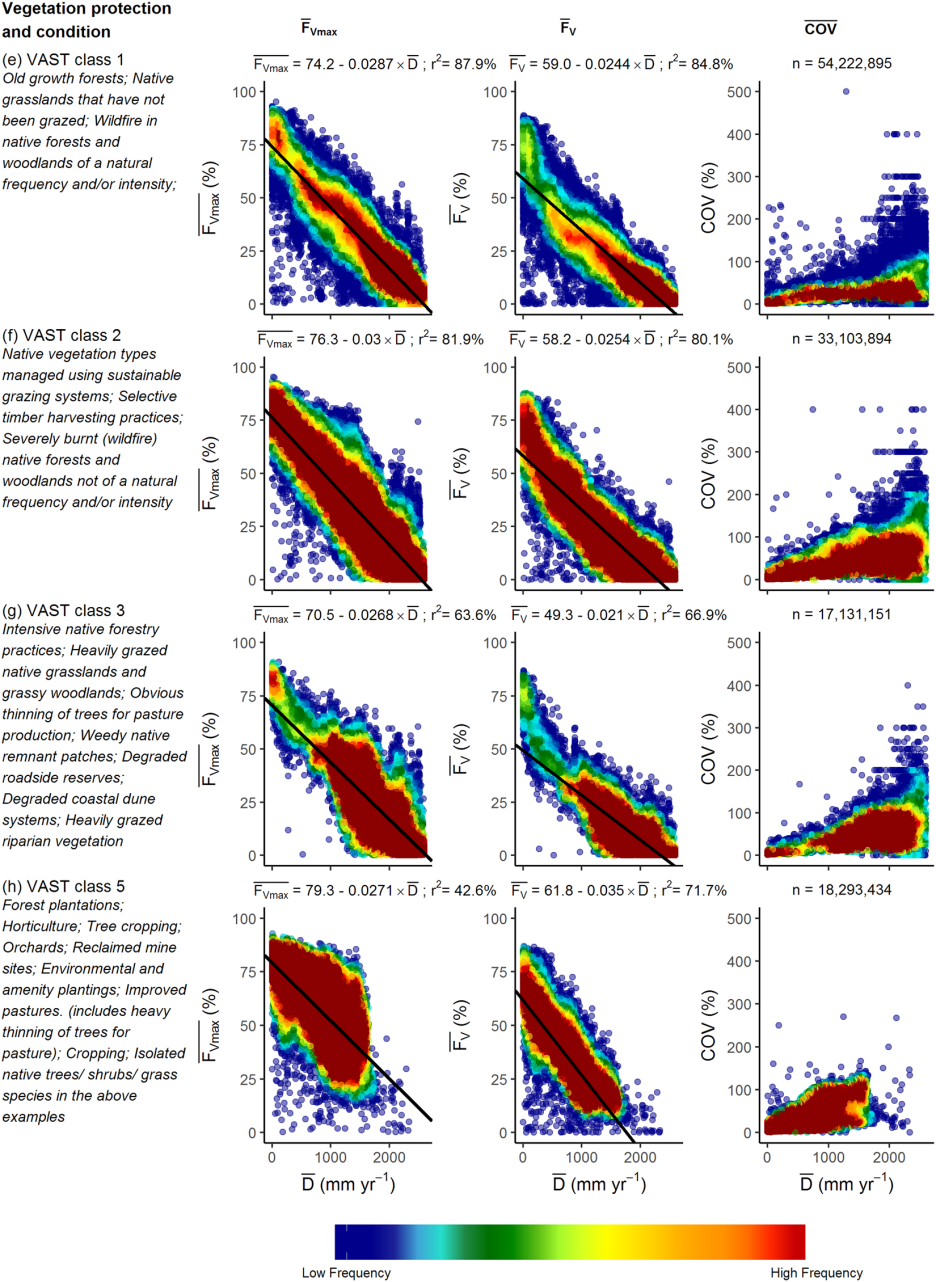
(**e**) Native vegetation in natural condition (VAST class 1); (**f)** Native vegetation modified by land use (VAST class 2); (**g**) Native vegetation transformed by land use (VAST class 3); (**h**) Native vegetation replaced with cultivated vegetation (VAST class 5).

**Figure 9:**
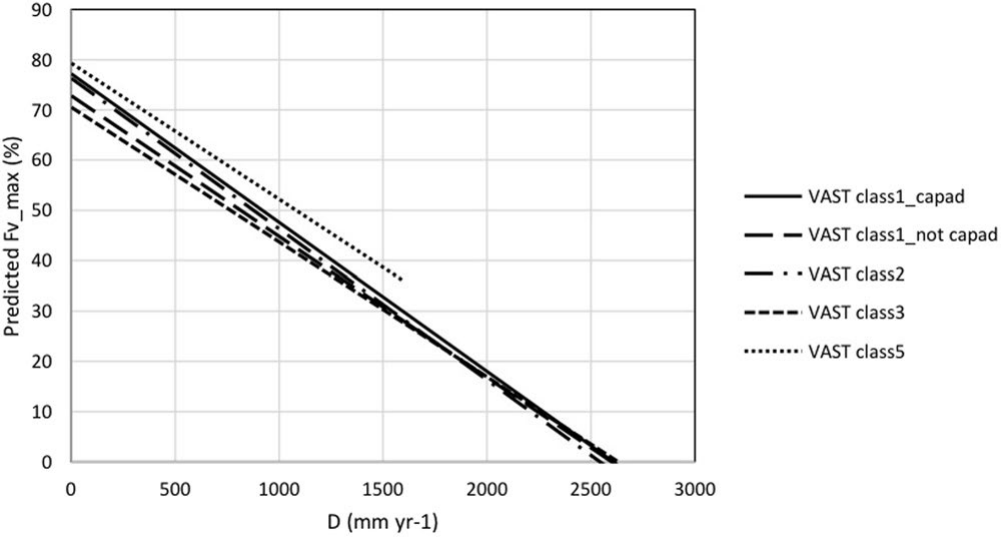
Comparison of linear least squares regression equations for predicting $\bar{<mml:mpadded xmlns:xlink="http://www.w3.org/1999/xlink" voffset="0">{F}_{vmax}</mml:mpadded>}$ from $\bar{D}$ (see Figs 7 and 8).

**Figure 10:**
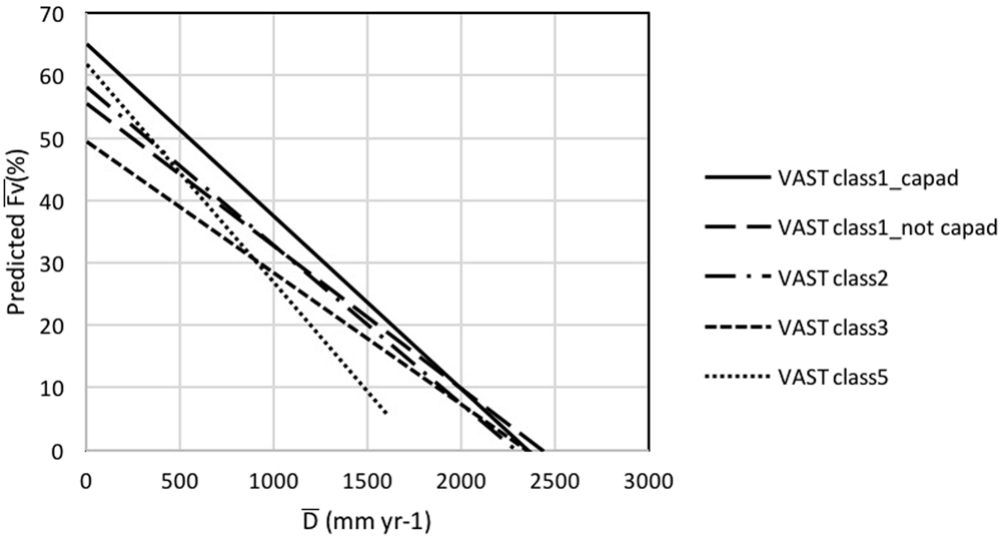
Comparison of linear least squares regression equations for predicting $\overline{{F}_{V}}$ from $\bar{D}$ (see Figs 7 and 8).

In Australia a subset of the vegetation that remains in a mostly natural condition is within protected areas that cover at the time of this analysis ~12% of the land area. The CAPAD 2008 spatial data layer includes both public and private lands with vegetation cover protected by a conservation agreement. Within the mapped protected areas, linear regression of $\bar{<mml:mpadded xmlns:xlink="http://www.w3.org/1999/xlink" voffset="0">{F}_{vmax}</mml:mpadded>}$ and $\bar{D}$ was able to account for 91% of the variance (Fig. 7a). When the dataset was further restricted to protected areas having intact native vegetation (VAST class 1), the variance accounted for by the linear regression relationship increased slightly to 92% (Fig. 7b). The regression equations for the relationships shown in Fig. 7a,b are almost identical. Outside of protected areas the variance accounted for by linear regression relationships (${\overline{F}}_{Vmax}$ and $\bar{D}$) of all VAST vegetation states and the subset of intact native vegetation were slightly reduced (r^2^ = 82% and 86% respectively; Fig. 7c,d). In Fig. 7a–d there appears to be an almost normal distribution of values of $\bar{<mml:mpadded xmlns:xlink="http://www.w3.org/1999/xlink" voffset="0">{F}_{vmax}</mml:mpadded>}$ about the regression line. That is not the case for the relationship between $\overline{{F}_{V}}$ and $\bar{D}$, where the data suggest a curvilinear relationship with a greater reduction in $\overline{{F}_{V}}$ with increasing $\bar{D}$. This is likely due to greater impacts of disturbances such as drought and wildfire with increasing evaporation deficit over the 10-year period of the dataset. Within protected areas the coefficient of variance of $\overline{{F}_{V}}$ for most pixels is less than 80% for $\bar{D}\le 2000$ mm yr^−1^ (Fig. 7a) and even lower when the dataset is restricted to intact native vegetation (Fig. 7b). This reflects the capacity of intact native vegetation to make optimal use of water over a decadal period. Outside of protected areas the COV of $\overline{{F}_{V}}$ is about twice that of vegetation within protected areas (Fig. 7c). The relationships between $\bar{<mml:mpadded xmlns:xlink="http://www.w3.org/1999/xlink" voffset="0">{F}_{vmax}</mml:mpadded>}$, $\overline{{F}_{V}}$, COV and $\bar{D}$ for the four VAST classes that we investigated are shown in Fig. 7e–h. Analyses of native vegetation in natural condition (VAST class 1; Fig. 8e) and native vegetation modified by land use (VAST class 2; Fig. 7f) yielded very similar linear regression relationships for $\bar{<mml:mpadded xmlns:xlink="http://www.w3.org/1999/xlink" voffset="0">{F}_{vmax}</mml:mpadded>}$ and $\bar{D}$, $\overline{{F}_{V}}$ and $\bar{D}$. The COV of many pixels in the VAST class 2 exceeded the values obtained in the VAST class 1 land use. That is to be expected as trees, which generally have the capacity to access water deep in the soil profile, have been to some extent removed or replaced by vegetation having a shallower rooting depth hence reduced access to water during dry periods. Within VAST class 2 low shrubs and herbaceous vegetation frequently is reduced by grazing of livestock, feral herbivores and/or large populations of kangaroos. Most of the pixels within VAST class 3 (native vegetation transformed by land use) occur in regions where $\bar{D} > 600$ mm yr^−1^ (Fig. 8g). Regression parameters differ from VAST class 2 largely due to the uneven spread of data points along the x-axis. Where native vegetation has been replaced with cultivated vegetation (VAST class 5; Fig. 7h) ${\overline{F}}_{Vmax}$ values tend to exceed those of the native vegetation. Within this VAST class pixels utilized for food and timber production likely receive inputs such as irrigation and nutrient addition. Herbaceous crops and pastures are commonly comprised of short-lived shallow-rooted vegetation. As water limitation increases, $\overline{{F}_{V}}$ of VAST class 5-replaced (managed) declines more rapidly than that of all other vegetation-land use classes considered here. To facilitate comparison between land-use change effects on predicted $\bar{<mml:mpadded xmlns:xlink="http://www.w3.org/1999/xlink" voffset="0">{F}_{vmax}</mml:mpadded>}$ and $\overline{{F}_{V}}$ the regression equations presented in Figs 7 and 8 are plotted together in Figs 9 and 10.

## Discussion and Conclusions

In this study we have shown that at a continental scale over a 10-year time period, the mean $\overline{{F}_{V}}$ of a high spatial resolution (250 m) time-series derived from MODIS NDVI data, is strongly positively correlated with modelled water availability index, $\bar{W}$, and strongly negatively correlated with modelled evaporation deficit, $\bar{D}$. The maximum mean annual *F*_*V*_, $\bar{<mml:mpadded xmlns:xlink="http://www.w3.org/1999/xlink" voffset="0">{F}_{vmax}</mml:mpadded>}$ is similarly strongly correlated with these climate parameters. For grid cells where the mean annual evaporation exceeds precipitation (i.e. water-limited conditions), least squares linear regression of $\overline{{F}_{V}}\,$($\bar{<mml:mpadded xmlns:xlink="http://www.w3.org/1999/xlink" voffset="0">{F}_{vmax}</mml:mpadded>}$) and $\bar{<mml:mpadded xmlns:xlink="http://www.w3.org/1999/xlink" voffset="0">{W}_{E}</mml:mpadded>}$ (Equation 3) accounted for 79% (81%) of the variance while the relationship between $\overline{{F}_{V}}\,$ ($\bar{<mml:mpadded xmlns:xlink="http://www.w3.org/1999/xlink" voffset="0">{F}_{vmax}</mml:mpadded>}$) and $\bar{<mml:mpadded xmlns:xlink="http://www.w3.org/1999/xlink" voffset="0">{W}_{Q}</mml:mpadded>}$ (Equation 2) accounted for 83% (78%). At the continental scale of our analyses, the finer spatial resolution of our MODIS-sourced dataset had no real effect on predictive capacity when compared to that of Berry and Roderick (Equation 5; 250 m versus 8 km pixels). The values of the regression coefficients, and the explained variance, are very similar to those obtained for $\overline{{F}_{V}}$ and $\bar{<mml:mpadded xmlns:xlink="http://www.w3.org/1999/xlink" voffset="0">{W}_{Q}</mml:mpadded>}$ calculated from the NOAA AVHRR NDVI time-series by Berry and Roderick^15,16^ and climate data estimates at 0.05 degree (~8 km) spatial resolution (see Equation 5). Least squares linear regression of $\overline{{F}_{V}}$ ($\bar{<mml:mpadded xmlns:xlink="http://www.w3.org/1999/xlink" voffset="0">{F}_{vmax}</mml:mpadded>}$) and $\bar{D}$ (Equation 7) accounted for 81% (84%) of the variance for the whole dataset.

Our results confirm that water and energy supply are the major determinants of vegetation greenness at the continental scale, irrespective of the vertical structure (i.e., the height, density, and layering) of the natural vegetation and its floristic composition. While both *W* and *D* are useful predictors of vegetation greenness from climate parameters, *D* is preferable for modelling potential future vegetation-climate effects as the relationship between *F*_*V*_ and *W* breaks down in energy limited environments while the relationship between *F*_*V*_ and *D* holds (*D* is always ≥0). However, calculating *D* requires *E*_*A*_, a variable that is not measured. Here we have calculated *E*_*A*_ at the scale of an individual spatial pixel of 250 m resolution using an empirical equation derived for water catchments. We recommend this as the best available approach, noting that it is inevitable there are trade-offs in the choice of model variables as a function of, among other things, data availability and the accuracy with which they represent bio-physical processes. We further note that our best value of *α* differed from that used for water catchment studies. That is to be expected as our study was restricted to vegetated areas. Runoff in vegetated areas is reduced due to increased interception by the canopy, increased evapotranspiration and impedance of storm flow by leaf litter and woody debris^38–44^.

Our results do not support our hypothesis that some of the scatter (i.e., actual $\overline{{F}_{V}}$ × predicted $\overline{{F}_{V}}$) in the study of Berry and Roderick may be due to the coarse (0.05 degree) spatial resolution of the dataset used by Berry and Roderick. Our results do suggest, however, that some of the scatter about the *F*_*V*_-*D* relationship is due to modification of the natural vegetation greenness from anthropogenic impacts on natural vegetation cover. This is supported by comparison of the linear regression equations for $\bar{<mml:mpadded xmlns:xlink="http://www.w3.org/1999/xlink" voffset="0">{F}_{vmax}</mml:mpadded>}$ and $\overline{{F}_{V}}$ representing the five land-cover types (Fig. 8). When the effects of random disturbances including wildfire, drought and timber harvesting within the decade of the study are minimised there is less than 10% variation in predicted $\bar{<mml:mpadded xmlns:xlink="http://www.w3.org/1999/xlink" voffset="0">{F}_{vmax}</mml:mpadded>}$ between intact native vegetation and transformed (heavily cleared) native vegetation. When conditions for plant growth are favourable the mean annual *F*_*V*_ of a grid cell will be similar regardless of the vegetation structure. When the impacts of disturbances are averaged out over a decade, however, mean decadal *F*_*V*_ of grid cells where native vegetation has been transformed by land use will likely be only ~75% of *F*_*V*_ of grid cells with identical *D* having protected, intact native vegetation cover. This proportionate reduction in decadal mean *F*_*V*_ is similar in magnitude to that predicted by Berry and Roderick^15^ for a reduction in atmospheric carbon dioxide concentration from 350 ppmv to 280 ppmv (representing the Pre-industrial Period).

It should be noted that this causal relationship was simply inferred, however, from the statistical correlations of *F*_*V*_ with land tenure and mapped vegetation condition. Within mapped vegetation condition class the degree of degradation is widely variable. We assumed that degrading impacts are excluded or less intense in protected areas. We consider these assumptions are reasonable given the extensive studies that have examined the impacts of specific land uses activities on land degradation^45,46^.

Nonetheless, there are other factors not examined here that effect the availability of plant water. We have assumed that the long-term average water balance of a grid cell is in equilibrium with the climate. On the Australian continent, in particular, the land surface and subsurface hydrological systems redistribute water over vast distances resulting in plant growth within climatically arid areas. Another common phenomenon in the arid zone is the access by deeply rooting perennial shrubs and trees to aquifer water^47^.

We also examined vegetation greenness in the absence of reference to structure or the floristic composition of the vegetation cover. Additional ecological and conservation insight can be gained from stratifying vegetation greenness time series data by vegetation structure and floristic information^31^. Furthermore, it is possible to infer vegetation structure from vegetation greenness time series data which holds potential for modelling past and future vegetation responses^15^.

An important application of vegetation greenness is its use as an input variable in models of gross primary productivity and simulations of terrestrial carbon dynamics^48^. While the relationship derived here between *F*_*V*_ and *D* is an empirical correlation, it is well-grounded in eco-physiology and bio-energetics and will therefore likely be sufficiently robust to be useful in simulating the impact of future climate on vegetation greenness. However, predicting vegetation in the future, or even the past, also requires inclusion of a model to account for impacts of CO_2_ concentration on vegetation growth^49^, along with projected changes in land use and its impact on vegetation condition.

## Electronic supplementary material

Additional Methods
