# Supplementary material for: On modelling the relationship between vegetation greenness and water balance and land use change
Source: Sci Rep. 2018 Jun 13;8:9066. doi: 10.1038/s41598-018-27139-0 (PMC5998125; doi:10.1038/s41598-018-27139-0)
Supplement: Supplementary file 1 — Additional Methods [file 41598_2018_27139_MOESM1_ESM.pdf]

Additional Methods

On modelling the relationship between vegetation greenness and water balance and land use change

Sandra L. Berry and Brendan Mackey*

Environmental Futures Research Institute, Griffith University, Gold Coast City, Australia
Griffith Climate Change Response Program, Griffith University, Gold Coast City, Australia

*Corresponding author (b.mackey@griffith.edu.au)

Preliminary NDVI data treatment

A 10-year time-series of MODIS 16-day L3 Global 250m (MOD13Q1) satellite imagery formed the base for our Fv (vegetation greenness) analyses. The spatially gridded NDVI layers for Australia were downloaded from https://remote-sensing.nci.org.au. A detailed description of the methodology used to produce these layers from the original tiles is provided by Paget and King (2008). The 230 images of the time-series we used represented the period 1 July 2000 to 30 June 2010. This time period was selected as it includes some of the wettest and driest years recorded in Australia (Bureau of Meteorology 2016).

Before analysing the NDVI data it was necessary to ensure that signal contamination (such as cloud) evident in the downloaded imagery was accounted for. This was achieved by sequentially analysing images comprising the time-series firstly for 'dropouts' and then for 'spikes' in reflectance values. A pixel value was identified as a 'dropout' if it had a value <90% of the value of the corresponding pixel in the previous and subsequent image. When a 'dropout' was detected, a value equal to the average of the values of the previous and subsequent image was substituted. A similar procedure was followed for the detection and correction of 'spikes'. Spikes were identified as having a value exceeding 110% of the corresponding pixels in the previous and subsequent image. The de-contaminated NDVI time-series data are subsequently referred to as $NDVI_{clean}$.

NDVI of bare soil, $NDVI_{soil}$

In order to identify pixels potentially lacking green vegetation cover we calculated spatially gridded minimum values of $NDVI_{clean}$ ($NDVI_{min}$) from the 230 images of the 10 year time-series. Raster layers, with spatial dimensions to match the NDVI imagery, were constructed for 25 major surface lithology groups represented in the digital database 'Surface geology of Australia 1:1,000,000 scale' (Raymond and Retter 2010). Frequency histograms of $NDVI_{min}$ were constructed for each lithology class, and the $NDVI_{min}$ value corresponding to the point

of maximum frequency was identified. This value was considered to be representative of $NDVI_{soil}$, the bare soil NDVI, for the lithological group. Values of $NDVI_{soil}$ for all 25 lithology groups (see Table 1) were then combined to produce a spatially gridded $NDVI_{soil}$ layer (Additional Methods Figure 1).

Hornfels and ultramafic lithologies do not occur in the arid zone, and volcanogenic sediments are rare. Consequently, for these classes, $NDVI_{min}$ would likely include green vegetation. We arbitrarily assigned $NDVI_{soil}$ of 0.20 to the hornfels and ultramafic lithologies. For the volcanogenic sediments, as a broad shoulder peak of the histogram of $NDVI_{min}$ had a mean frequency of 0.17, we equated this with $NDVI_{soil}$. $NDVI_{soil}$ for the lake and swamp deposits lithology class was estimated at 0.03. Such a low value was expected as this lithology class includes playa lakes that commonly have a salt crust. For 93% of pixels $NDVI_{soil}$ had a value between 0.13 and 0.17, supporting the selection of a continental value of 0.15 used by (Donohue et al. 2013).

Additional Methods Figure 1. Frequency histogram of estimated $NDVI_{soil}$ for Australia.

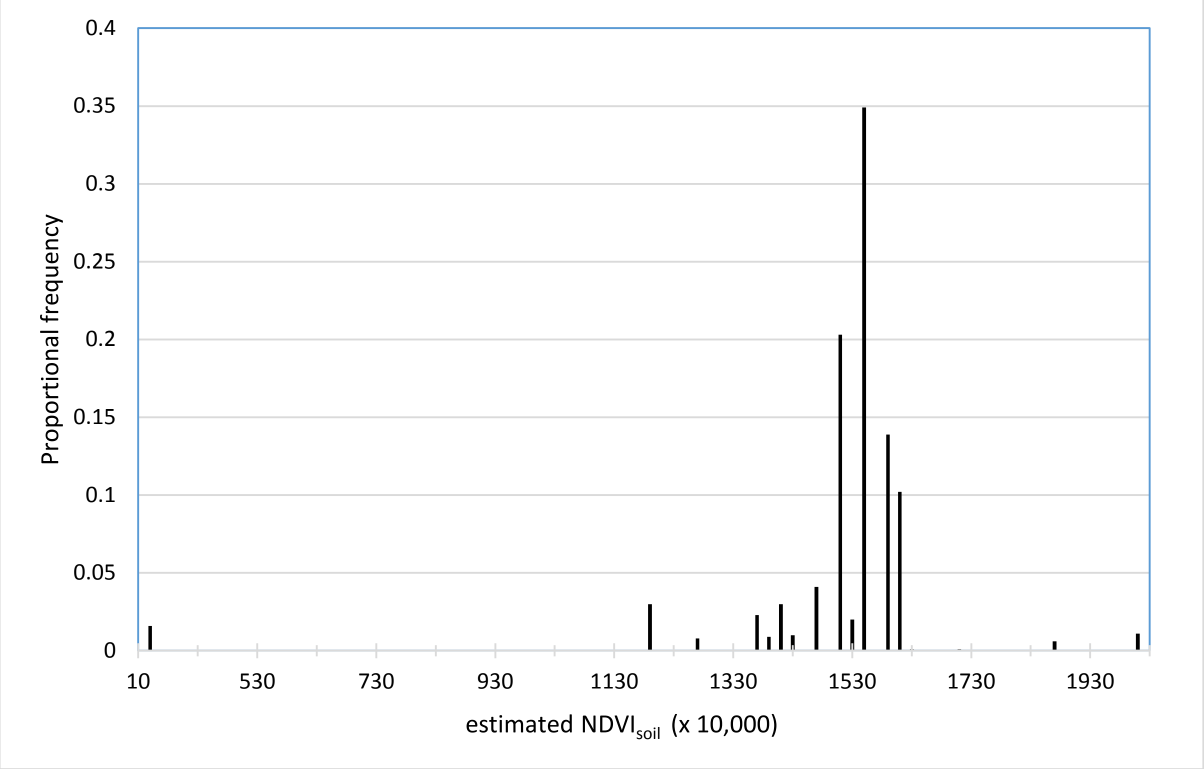

Additional Methods Table 1. Estimated NDVI of bare soil ($NDVI_{soil}$) for major lithological classes over Australia.

Lithology class (including surface lithology code provided in the digital database)	Number of pixels within lithology class	$NDVI_{soil}$ ×10000 (estimated value for bare soil in parentheses)	Frequency of pixels at $NDVI_{soil}$	Comments
Alluvia – a	12762705	1610	102017	
Calcrete - k	1262602	1550	25646	
clay- sandstone - c	3441653	1190	25894	sedimentary siliclastic
Colluvium - r	16289900	1590	183990	
detrital mafic various- w	999924	1270	5366	
duricrust carbonate - l	3798661	1410	33820	
dunes - d	25327417	1510	471153	
estuarine - e	633168	1860	2369	
felsic intrusive - g	5026349	1460	39526	
hornfels - h	9978	3140 (2000)	86	none in arid zone
felsic volcanic - f	1179008	1580	5501	
igneous metasedimentary - x	96943	1460	1059	
volcanogenic sediment - j	73484	2600 (1700)		rare in arid zone
lake, swamp deposits - t	1663216	340	11453	
marine - i	2553912	1530	64747	
metamorphic - m	86766	1630	1015	
metamorphic – n	1236631	1420	16220	
metasedimentary – y	1114826	1390	6464	
organic – o	553569	2010	4394	
quartz spring – q	4013	970	89	
sedimentary sandplain – s	42617281	1550	392085	
silcrete – z	263577	1180	3546	
ultramafic – u	818947	2770 (2000)	5186	not in arid zone
volcanic – b	2917669	1370	12590	
volcanic – v	57935	1220	970	

F_V

We calculated a time-series of F_V, the fraction of photosynthetically active radiation intercepted by the sunlit canopy from the SAVI time-series using a linear transform (Carlson and Ripley 1997, Roderick et al. 1999, Donohue et al. 2013) (Additional Methods Figure 2):

$$F_V = \frac{NDVI_{clean} - NDVI_{soil}}{NDVI_{max} - NDVI_{soil}} \qquad \text{Eqn 3}$$

Where $NDVI_{max}$ has a value of 1.00.

For each of the ten years of the time-series we calculated monthly values of F_V. It was assumed that F_V of a 16-day composite image represents an average value for the 16-day period. The monthly values of F_V were calculated using a weighting for the number of days of each 16-day compositing period that occur in each month. An annual mean, $\overline{F_{V-yr}}$, was calculated for each of the ten years of the time-series. The mean F_V for the 10-year time-series is subsequently referred to as $\overline{F_V}$.

Several large scale disturbances, including extensive forest fires in south-eastern Australia and drought and cyclone damage to tropical rainforests in north-eastern Australia, impacted on the vegetation during the period of this study. As an aim of this study was to assess the predictive capacity of environmental variables we needed to minimize the signal of extraneous noise arising from random environmental events such as fire, cyclones and floods. To achieve this we determined the maximum value of $\overline{F_{V-yr}}$. This spatial layer is subsequently referred to as $\overline{F_{Vmax}}$. We also determined the minimum value of $\overline{F_{V-yr}}$, which we refer to as $\overline{F_{Vmin}}$

Additional Material Figure 1. Gridded estimates of mean fraction of photosynthetically active radiation absorbed by the sunlit canopy ($\overline{F_V}$) for the Australian continent.

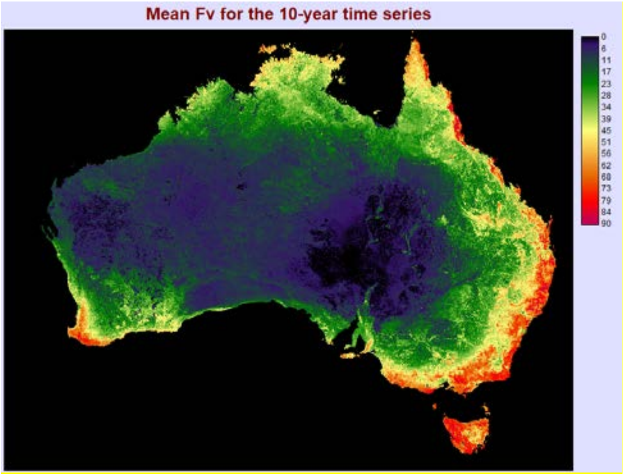

Climate parameters

Gridded surfaces of monthly rainfall, pan evaporation and global radiation were generated by the MTHCLIM component of the ANUCLIM software package (Xu and Hutchinson 2013) and ground-level elevation data from the GEODATA 9 second DEM (Hutchinson et al. 2008). The monthly mean climate surfaces of precipitation (P, mm) and pan evaporation (E_{pan}, mm) were generated for the period 1976-2005. Monthly means of daily global solar radiation on rainfall (Q_S, MJ m^{-2} day^{-1}) were calculated for the period 1970-1995. Mean annual rainfall, $\bar{P}$, and mean annual pan evaporation, $\overline{E_{pan}}$, were calculated by summing the grids of the respective monthly means. Mean annual $\overline{Q_s}$ was calculated by summing the monthly mean daily radiation and multiplying the sum by 30. Potential evaporation, $\overline{E_P}$, was derived from $\overline{E_{pan}}$ (Hobbins, Dai et al. 2008):

$$E_p = kE_{pan} \qquad \text{Eqn 4}$$

where the constant, k, has a value of 0.75 for Australian evaporation pans.

Two estimates of W were calculated:

$\overline{W_Q} = \bar{P} - \overline{Q_s}/\lambda$ (see Eqn 2) where λ is (~2.5×10^6 J kg^{-1} H_2O).

$$\overline{W_E} = \bar{P} - \overline{E_P} \qquad \text{(Eqn 5)}$$

The formulation of Choudhury (1999) for catchments was used to estimate actual evaporation, $\bar{E}$ (mm yr^{-1});

$$\bar{E} = \frac{\bar{P}}{[1+(\frac{\bar{P}}{\overline{E_P}})^{\alpha}]^{1/\alpha}} \qquad \text{Eqn 6}$$

where α is a ‘catchment properties parameter’ that alters the partitioning of P to E_P and runoff. A range of values of α were applied to calculate $\bar{D}$;

$$\bar{D} = \overline{E_P} - \bar{E}$$

as visualised in Additional Methods Figure 2.

Figure A.M. 2 Visualisation of gridded values of $\bar{D}$ for the Australian continent based on α values of 6 and 1.9.

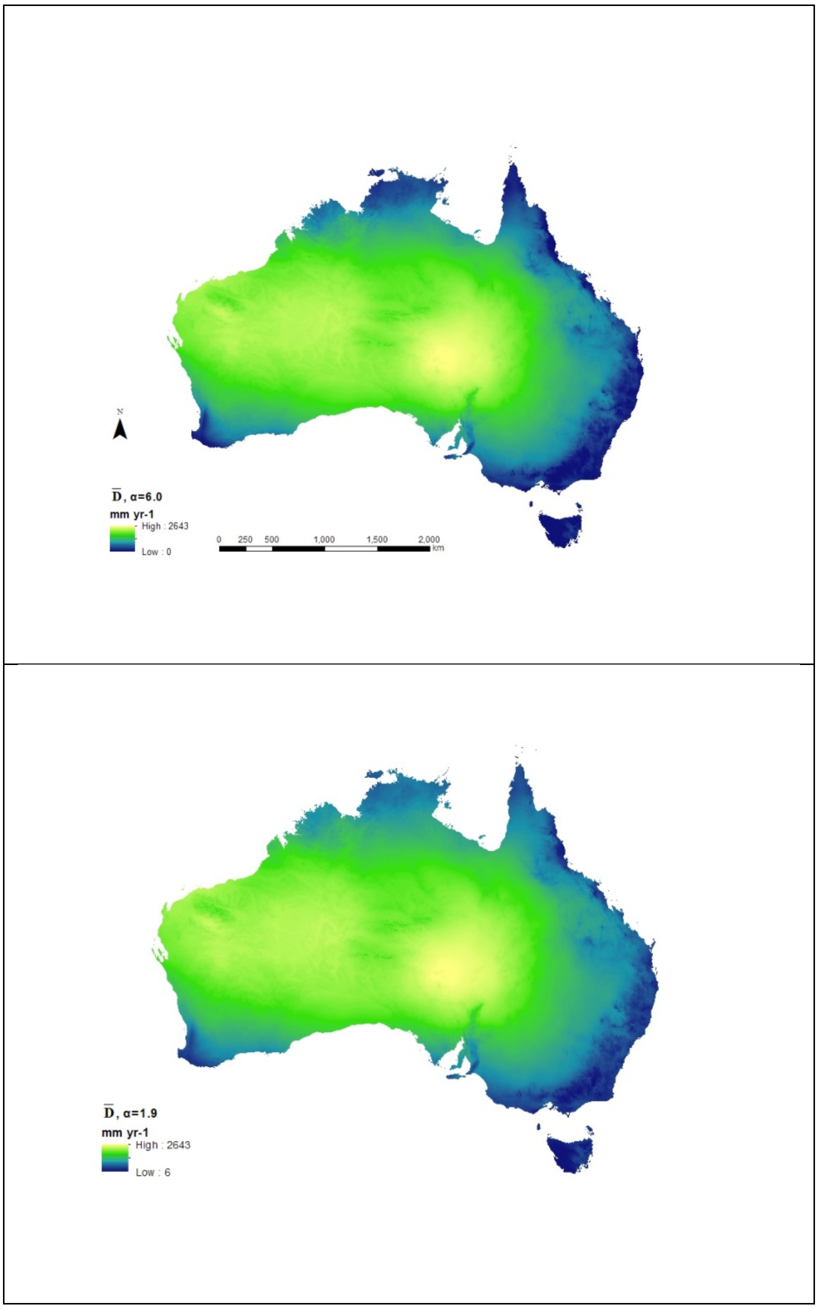
N
$\overline{D}$, α=6.0
mm yr-1
High : 2643
Low : 0
0 250 500 1,000 1,500 2,000
km
$\overline{D}$, α=1.9
mm yr-1
High : 2643
Low : 6

Additional references

Carlson, T. N. and D. A. Ripley (1997). "On the relation between NDVI, fractional vegetation cover, and leaf area index." Remote Sensing of Environment **62**(3): 241-252.

Choudhury, B. J. (1999). "Evaluation of an empirical equation for annual evaporation using field observations and results from a biophysical model." Journal of Hydrology **216**: 99-110.

Donohue, R. J., et al. (2013). "Impact of CO_2 fertilization on maximum foliage cover across the globe's warm, arid environment." Geophysical Research Letters **40**: 1-5.

ESRI (2012). ArcGIS 10.2; http://www.esri.com/software/arcgis/arcgis-for-desktop

Hutchinson, M. F., et al. (2008). Geodata 9 second DEM and D8 User Guide. T. A. N. U. a. G. A. Fenner School of Environment and Society. Canberra, Geoscience Australia.

Clark Labs (2009). IDRISI: The Taiga Edition. Worcester, MA, Clark University; https://clarklabs.org/download/

Bureau of Meteorology (2016) Climate change and variability; http://www.bom.gov.au/climate/change/index.shtml#tabs=Tracker&tracker=timeseries .

Paget, M. J. and E. A. King (2008). MODIS Land data sets for the Australian region. CSIRO Marine and Atmospheric Research Internal Report 004. Canberra, CSIRO Marine and Atmospheric Research. **004:** 96.

Raymond, O. L. and A. J. Retter (2010). Surface geology of Australia 1:1,000,000 scale [digital dataset]. C. o. A. Geoscience Australia. Canberra.

Roderick, M. L., et al. (1999). "Estimating woody and herbaceous vegetation cover from time series satellite observations." Global Ecology and Biogeography Letters **8**: 501-508.

Xu, T. and M. F. Hutchinson (2013). ANUCLIM version 6.1. Fenner School of Environment and Society. The Australian National University, Canberra; http://fennerschool.anu.edu.au/research/products/anuclim-vrsn-6
